# FaceGuard: A Wearable System To Avoid Face Touching

**DOI:** 10.3389/frobt.2021.612392

**Published:** 2021-04-08

**Authors:** Allan Michael Michelin, Georgios Korres, Sara Ba’ara, Hadi Assadi, Haneen Alsuradi, Rony R. Sayegh, Antonis Argyros, Mohamad Eid

**Affiliations:** ^1^ Applied Interactive Multimedia Lab, Engineering Division, New York University Abu Dhabi, Abu Dhabi, United Arab Emirates; ^2^ Clinical Associate Professor, Cornea and Refractive Surgery, Cleveland Clinic Abu Dhabi, Abu Dhabi, United Arab Emirates; ^3^ Professor at the Computer Science Department (CSD), University of Crete (UoC), Crete, Greece

**Keywords:** face touching avoidance, IMU-based hand tracking, sensory feedback, vibrotactile stimulation, wearable technologies for health care

## Abstract

Most people touch their faces unconsciously, for instance to scratch an itch or to rest one’s chin in their hands. To reduce the spread of the novel coronavirus (COVID-19), public health officials recommend against touching one’s face, as the virus is transmitted through mucous membranes in the mouth, nose and eyes. Students, office workers, medical personnel and people on trains were found to touch their faces between 9 and 23 times per hour. This paper introduces FaceGuard, a system that utilizes deep learning to predict hand movements that result in touching the face, and provides sensory feedback to stop the user from touching the face. The system utilizes an inertial measurement unit (IMU) to obtain features that characterize hand movement involving face touching. Time-series data can be efficiently classified using 1D-Convolutional Neural Network (CNN) with minimal feature engineering; 1D-CNN filters automatically extract temporal features in IMU data. Thus, a 1D-CNN based prediction model is developed and trained with data from 4,800 trials recorded from 40 participants. Training data are collected for hand movements involving face touching during various everyday activities such as sitting, standing, or walking. Results showed that while the average time needed to touch the face is 1,200 ms, a prediction accuracy of more than 92% is achieved with less than 550 ms of IMU data. As for the sensory response, the paper presents a psychophysical experiment to compare the response time for three sensory feedback modalities, namely visual, auditory, and vibrotactile. Results demonstrate that the response time is significantly smaller for vibrotactile feedback (427.3 ms) compared to visual (561.70 ms) and auditory (520.97 ms). Furthermore, the success rate (to avoid face touching) is also statistically higher for vibrotactile and auditory feedback compared to visual feedback. These results demonstrate the feasibility of predicting a hand movement and providing timely sensory feedback within less than a second in order to avoid face touching.

## 1 Introduction

Coronavirus disease (COVID-19), caused by severe acute respiratory syndrome coronavirus 2 (SARS-CoV-2), has spread worldwide, with more than 88 million cases and 1.9 million fatalities as of January, 2021 WHO (2020). Maintaining social distancing, washing hands frequently, avoiding touching the face including eyes, nose, and mouth, are the major methods associated with preventing COVID-19 transmission Chu et al. (2020). Contaminated hands have the potential to disseminate COVID-19 especially if associated with touching the face Macias et al. (2009). Face touching is an act that can happen without much thought, and in fact, happens with such a high occurrence that reducing it could mitigate a heavy source of transmission. Beyond simple skin irritations, face touching has been linked to emotional and cognitive processes Barroso et al. (1980), Mueller et al. (2019), increasing with attentiveness while tasks are being performed, as well as with increasing pressure and anxiety Harrigan (1985). For such common underlying motives, it is no surprise to see that on average a person touches their face 23 times in an hour Kwok et al. (2015). Given that the primary source of COVID-19 transmission is through contact with respiratory droplets [via the nose, mouth, or eyes, either directly from another individual or picked up from a surface Pisharady and Saerbeck (2015)], avoiding face touching is of a great value.

Developing a system to avoid face touching outright by stopping hand movement raises two main challenges. First of all, a system must predict rather than detect when a hand movement will result in face touching well before the hand reaches the face. Secondly, once a hand movement is predicted to result in face touching, a sensory feedback must be presented immediately in order to stop the hand movement and thus avoid face touching. Note that the prediction and response components are evaluated separately to better analyze the capabilities/limits of each component.

### 1.1 Predicting Hand Movement

Predicting face touching requires precise hand tracking. Two common approaches for tracking hand movement are vision-based approaches Al-Shamayleh et al. (2018) and wearable sensor-based approaches Jiang X. et al. (2017), Mummadi et al. (2018). A combination of these have also shown potential for enhanced accuracy Jiang S. et al. (2017), Siddiqui and Chan (2020). Vision-based hand tracking utilizes camera networks Pisharady and Saerbeck (2015), and as mentioned, can be supplemented with wearable devices such as motion sensor systems placed along the body, to map either whole body or hand movement Liu et al. (2019). One particular wearable device often used is the inertial measurement unit (IMU), capable of collecting data along six degrees of freedom, with three additional angular sensors to enable a total of nine inputs. Found in many smart watches, the IMU is equipped with an accelerometer and gyroscope, providing an inexpensive option that is not only accurate, taking measures along all three dimensions for each of its components, but also one that does not require complementary infrastructure to operate. This allows the IMU to be versatile yet effective in the context in which it is implemented.

Paired with an appropriate machine learning model, the data from an IMU can be used to notify a user how often they are touching their face, as well as whether they have done so after each movement. IMUs have been used to correctly identify a completed face touch with high accuracy Fu and Yu (2017), Rivera et al. (2017). Even though detecting face touching greatly supports awareness training, it does not prevent face touching from happening. The motivation of the proposed system is to apply machine learning in order to predict face touching and provide vibrotactile feedback to prevent it rather than detecting it.

### 1.2 Sensory Feedback for Motor Control

Along with the development of hand tracking, the user must also be notified of their impending action before it is committed, with ample time for them to react. The notification must be delivered through a medium that will elicit the fastest response time. The three feedback modalities of relevance are visual, auditory, and vibrotactile, and it has been shown that vibrotactile feedback produces the fastest response times Ng and Chan (2012). Vibrotactile feedback systems can be used to achieve this, with benefits similar to that of an IMU, being cost-effective, and easily implemented into a wearable device.

A low-cost wearable system that prevents people from touching their face, and in the long run, assist people in becoming more aware of their face-touching, is proposed. The system exploits widespread and off-the-shelf smartwatches to track the human hand and provide timely notification of hand movement in order to stop touching the face. The decision to build the system with just a smartwatch makes it immediately available to people, without the requirement of building or wearing additional hardware. The system assumes a smartwatch with an IMU module and a vibration motor; a reasonable assumption as most commercial smartwatches are equipped with such hardware. Although preventing the spread of COVID-19 is the most evident, the system can be adapted for other applications such as habit reversal therapy (HRT) Bate et al. (2011) and treatment of chronic eye rubbing McMonnies (2008). The main contributions of this paper are summarized as follows:

1. Proposing a conceptual approach that utilizes IMU data to predict if a hand movement would result in face touching and provides real-time sensory feedback to avoid face touching.
2. Developing a model for tracking hand movement and predicting face touching using convolutional neural networks based on IMU data. To train the model, a database of 4,800 hand motion trials recording with 40 users under three conditions, sitting, standing, and walking is built.
3. Presenting a psychophysical study with 30 participants to compare the effectiveness of sensory feedback modalities, namely visual, auditory, and vibrotactile, to stop the hand while already in motion before reaching the face. The response time and success rate were used as the evaluation metrics for the comparison.

## 2 Related Work

### 2.1 Understanding Hand Movement

The detection and classification of body activity is a major area of research, with applications and techniques ranging from wearable electrocardiogram recorders to classify body movements in patients with cardiac abnormalities Pawar et al. (2007), recognition and 3D reconstruction of the face using computer vision Chen et al. (2017), Zhao et al. (2018), Lv (2020), Yang and Lv (2020), to activity tracking of remote workers through sensory systems Ward et al. (2006), Manghisi et al. (2020). For instance, a system named HealthSHIELD utilized Microsoft Kinect Azure D-RGB camera to detect high/low risk face touching in order to monitor compliance with behavioral protection practices. Results demonstrated an overall accuracy of 91%. Inertial Measurement Units (IMU) are another particularly common alternative that although can be used in tandem with other systems Corrales et al. (2008), can provide exceptional results on its own Olivares et al. (2011).

In connection to real-time hand movement recognition in virtual reality games, a wearable IMU has been investigated as an alternative to simple button presses on a controller to identify player action intent Fu and Yu (2017). Similar to the IMU implementation of our own study, an accelerometer, gyroscope, and magnetometer are used as the sensor inputs for classification. Once a user moves their hand in a predetermined pattern, a trained long short term memory (LSTM) model identifies the movement, and the relevant in-game controls are carried out.

Detecting the touching of one’s face using an IMU has been examined recently Christofferson and Yang (2020). A convolutional neural network is used to identify whether a user had touched their face at the end of a gesture. Once a user made their move, the collected data from the nine data modes of the IMU are passed through a trained model, with a face touch classification provided simply as true or false. This approach resulted in a 99% accuracy rate.

As is seen in previous studies, the deep learning model used alongside the IMU varies. Requiring a time series based solution, recurrent neural networks, particularly LSTM, and convolutional neural network (CNN) models have been implemented with significant success Rivera et al. (2017), Christian et al. (2019). Combinations of CNN layers with LSTM models have also been effective in processing IMU data Silva do Monte Lima et al. (2019). However, in related works where classification time is relevant, a standalone CNN has shown great promise Huang et al. (2017).

### 2.2 Real-Time Sensory Feedback

In order to provide a real-time sensory feedback to stop the hand movement and avoid face touching, multiple feedback sensory modalities can be utilized. Sensory feedback is usually presented through visual, auditory, and tactile modalities. Visual modality stimuli such as flashing is common in several warning systems, such as road transport industries Solomon and Hill (2002) and crosswalk warning systems Hakkert et al. (2002). In addition to the use of vision, auditory modality is widely used in transport, heath care, and industrial environments as it has an immediate arousing effect Sanders (1975). For instance, a previous study showed that auditory alarms used in helicopter environments conveyed urgency Arrabito et al. (2004). Comparing the two modalities, it was found that the response time to visual and auditory stimuli is approximately 180–200 and 140–160 ms, respectively Thompson et al. (1992). This is based on a previous finding that an auditory stimulus takes only 8–10 ms to reach the brain whereas visual stimulus takes 20–40 ms Kemp (1973). However, there are several factors that influence the average human response time include age, gender, hand orientation, fatigue, previous experience, etc. Karia et al. (2012).

Vibrotactile modality has also been found to improve the reaction time for several applications such as drone tele-operation Calhoun et al. (2003), Macchini et al. (2020), collision avoidance while driving Scott and Gray (2008), and alteration of motor command in progress (such as altering a reach in progress) Godlove et al. (2014). The temporal aspects of visual and vibrotactile modalities, as sources of feedback about movement control, are examined in Godlove et al. (2014). A modified center-out reach task where the subject’s hand movement was occasionally interrupted by a stimulus that instructed an immediate change in reach goal is utilized. Results demonstrated that the response for tactile stimuli was significantly faster than for visual stimuli.

Utilizing vibrotactile feedback for alarming the user about face touching has recently been studied. A commercial product, named IMMUTOUCH, utilized a smart wristband that vibrates every time the user touches their face Immutouch (2020). A recent research study presented a wearable system that utilizes a smartwatch to provide vibrotactile feedback and a magnetic necklace to detect when the hand comes to a close proximity to the face D’Aurizio et al. (2020). Even though these solutions are a great step forward to reducing the number of face touches and their duration, they do not consider real-time touch avoidance. Furthermore, these studies did not perform any systematic studies to determine the most effective sensory feedback modality to stop the hand movement and eventually avoid face touching. Aside from differences in the type and architecture of the deep learning model used for classification, our study employs a wearable IMU not just to classify a gesture, but to predict a gesture before it happens. The motion input data therefore will not include the final portion of an individual’s hand movement, placing a limit on the available data for training. In examining feasibility of success under such constraints, optimal sensory feedback thus plays a significant role.

## 3 Proposed Approach

**FIGURE 1:**
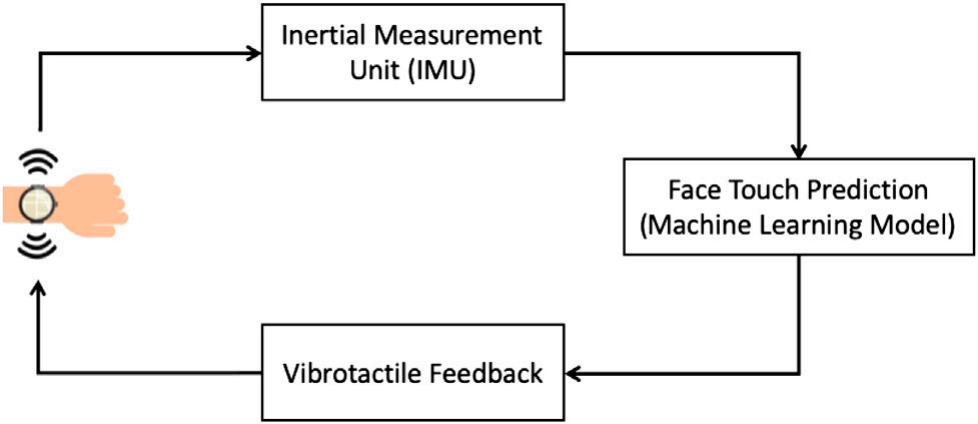
The application scenario involves a smartwatch with Inertial Measurement Unit to detect hand movement, a machine learning model to predict when a movement results in touching the face, and a vibration motor to alert the user in order to stop the hand movement.

A high-level description of the system is visualized in Figure 1. The system utilizes IMU data to measure hand movement, convolutional neural networks to predict, in real time, whether a hand movement will involve touching the face, and vibrotactile feedback to alert the user so they stop their hand movement before touching their face. Note that the system must perform in real time in order to generate response to stop the hand movement before it reaches the face.

**FIGURE 2:**
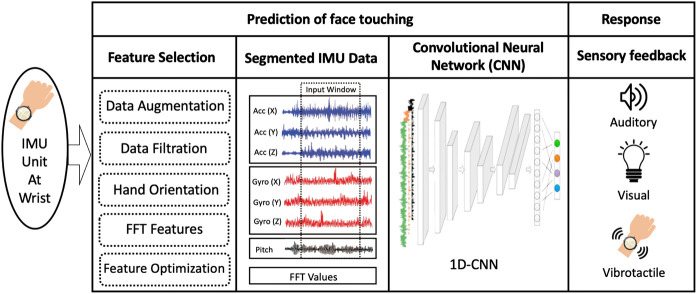
Overview of the FaceGuard system. Abreviations: FFT (Fast Fourier Transform), IMU (Inertial Measurement Unit), CNN (Convolutional Neural Network).

A more detailed description of the system is shown in Figure 2 while a technical description of the system is further analyzed in Section 4. The prediction component involves a sequence of three processes, namely feature selection, data segmentation, and a Convolutional Neural Network (CNN). Three sensory feedback modalities are considered for the response component, namely visual, auditory, and vibrotactile. Section 5 presents a psychophysical experiment to compare these modalities and inform the decision about using vibrotactile feedback.

A wearable device with an embedded IMU recording nine different types of hand motion data (*x*, *y*, and *z* components for accelerometer and gyroscope, and rotational pitch, roll, and yaw) makes the input to the prediction component. In the feature selection process, features are extracted and evaluated for relevance to predicting face touching hand movement. These features are used to improve the performance of the prediction model. Feature selection included several data pre-processing procedures such as data augmentation (to increase the size of training data), data filtration to enhance the signal-to-noise ratio, hand orientation calculation, Fast Fourier Transform (FFT) features extraction, and optimization of the combined features.

Once the features are identified, the time-series of the selected features are segmented according to a time window. The window size is an extremely important parameter to optimize in this process since it controls the tradeoff between response time and prediction accuracy. Once the time series data are segmented, all the features are fed into a one dimensional convolutional neural network (1D-CNN) model. 1D-CNNs are generally excellent in automatically detecting temporal relationships in multi-channel time-series data with minimal feature engineering. Using the 1D-CNN kernels allows an automatic extraction of the temporal features in IMU data, which is deemed important in recognizing hand movement towards the face through its corresponding IMU data. The model is trained and evaluated with data generated for this purpose that is recorded from 40 participants. Each participant went through a data collection session that consisted of two runs. In each run, the participant had to perform 10 face-touching hand movements during each of the following everyday activities (standing, walking, sitting) as well as 10 non-face touching hand movements during the same activities. Thus, each participant contributed 120 trials, yielding a total of 4,800 trials. The CNN model provides a binary output, whether the respective hand movement is predicted to result in face touching or not.

As soon as a prediction of face touching event is made, the response component renders a sensory feedback to alert the user, while the hand is in motion, to immediately stop the hand movement in order to avoid face touching. Based on the findings of Section 5, vibrotactile feedback is utilized as the sensory feedback modality as it provided superior performance (measured using the response time and success rate of avoiding face touching), compared to visual or auditory.

## 4 Prediction of Face Touching

### 4.1 Data Collection

The data collection procedure combines computer and smartwatch interfaces to collect the needed participant data. The hardware used to collect the IMU data is an Esp32-powered, M5Stack development watch known as M5StickC. It has six degrees of freedom consisting of a 3-axis accelerometer and a 3-axis gyroscope, with pitch, yaw, and roll being calculated internally.

**FIGURE 3:**
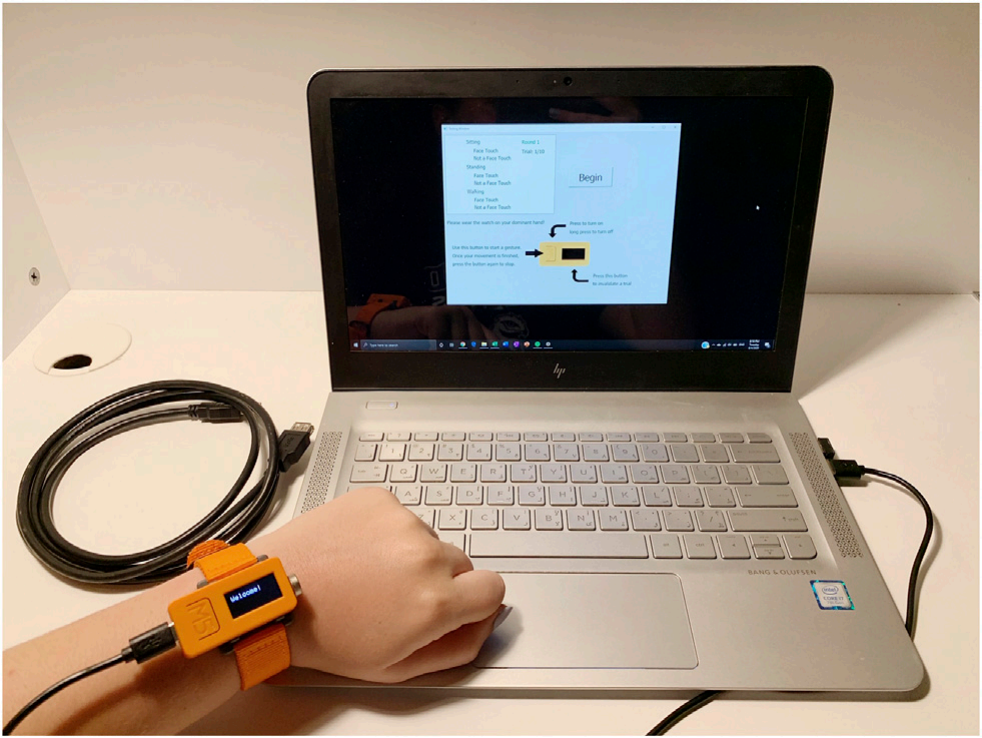
The general setup of the participation. The user is provided with a Windows laptop, the M5StickC watch, a USB-C cable, as well as a USB extension cable to be used for the walking trials.

Using an Arduino IDE, the M5StickC is programmed to read the IMU data and store it into a file through a serial connection to a computer. It relies on input from two buttons: the main button used to start and stop the recording of IMU data, and the side button used for user error correction related to trial invalidation and repetition. The program runs for a predetermined number of trials per session before sending an end signal to the computer that saves all the data to a file. The participation protocol in a session consists of two runs, with 60 trials each (30 face-touching and 30 non-face touching hand movements), that are repeated twice to gather a total of 120 trials. As can be seen in Figure 3, *sitting*, *standing*, and *walking* are considered as the three main activity types due to them being the most common positions taken in our daily lives. Thus, gathering data for *touching* and *not touching the face* for each of those stances would allow the trained model to make accurate predictions regardless of the user’s position.

The M5StickC is used in conjunction with a computer GUI application developed to ensure a holistic, user-friendly collection protocol. Its main purpose is to guide users through the participation and to store auxiliary user information that may be useful in optimizing the prediction model, including height, arm length, and age group. Users are first asked to fill out the aforementioned optional information fields. Then, the application window displays a list of the different sessions to be completed and their associated number of trials, with instructions on the watch’s hardware as well as the next steps. Both interfaces rely on a communication of signals to control the start and end of the data collection process. The M5StickC starts recording the moment the main button on the watch is pressed and stops recording when the same button is pressed again by the participant. A single gesture is recorded in this fashion.

The data are then stored into a file. To prevent the loss of data that may occur if the serial connection is interrupted, the user is provided with the option to save their data at any point during the participation upon exiting the computer application.

The general protocol for collecting the data relies on remote participation in compliance with global social-distancing and safety procedures. Users first receive a consent form and statement containing information and instructions pertaining to the participation. If consent is provided, they are given the watch, a compatible laptop, and all needed accessories to complete the required number of sessions in their own homes, as can be seen in Figure 3. The equipment is then sanitized properly before being passed on to the next participant. To ensure overall user anonymity, no identifying information is asked for or stored. Additionally, the protocol is asynchronous, which provides users with the freedom to complete the participation at their own pace as it is not compulsory to complete all trails and sessions in one run, rather users are encouraged to take a break at any point and return later to finish.

Overall, 40 sessions were recorded by 40 participants collecting 4,800 trials in total as elaborated in Section 3. Of the information disclosed to us, 15 of the 40 participants were female, 15 were male, and 10 undisclosed. Additionally, most of the participants were young adults, with the most common age range being 16–20 years followed by 21–25 years.

### 4.2 Data Preparation and Inputs

Once data collection is completed, data are prepared for the training and testing of the CNN-based prediction model. Each gesture lasts varying amounts of time, and therefore, requires a select window size to ensure prediction before a face touch has occurred. However, during data collection, users are able to begin their gestures at any point once the start button has been pressed. As such, each trial recording includes a static component (the duration before the hand movement starts), potentially shifting relevant data outside of the determined window. A script that produces plots displaying averaged sensor values over time identifies the lengths of these gestures. The script is applied to each file individually, providing plots for each feature (IMU sensory data)—split into sub plots for each stance (sitting, standing, and walking). The lengths of the static component of every plot at the beginning of the gesture are recorded and averaged, with the resulting values to be referenced for trimming during data preparation. These plots are also used to observe data trends among each feature. It is observed that the roll and yaw did not yield a discriminative pattern for the hand touch condition and thus they are excluded from the analysis. Further confirmation is obtained during the training process of the model; removing these two features improved the accuracy of the model. Furthermore, it is observed that it takes around 1,200 ms to complete a hand movement that involves face touching, which marks the upper limit for the total response time of the proposed system (prediction and motor response).

From the total number of gestures (4,800), the training and testing data sets are formed, randomly split 80–20% (3,840/960 gestures), respectively, and the two 3D input matrices are constructed. Splitting was done by participants; data from a single participant exist either in the training or the test set. This is to ensure the model is resilient to behavioral differences among participants. Filtration is also undergone, where gestures that finish before reaching the time required for the allotted window size are removed. In other words, gestures with very short duration (shorter than the window size of the 1D-CNN) are omitted from the dataset.

One challenge for developing a robust prediction model comes from the lack of large-scale data samples (40 participants with 120 trial repetition). To overcome this problem, data augmentation is introduced to prevent overfitting and improve generalization of the model. Augmentation is done by creating copies of the training data set and shifting it in time with ‘N’ number of steps while maintaining a constant window size. Augmentation is a great tool for populating the training data such that they share the same characteristics of the original set (representing the events of touching or not touching the face).

Frequency domain signature of hand movement toward the face can be obtained by taking the Fourier transform of the chosen IMU signals. Frequencies of noise can be learnt and discarded once the frequency domain features are obtained. The fast Fourier transform algorithm which is readily available in NumPy library in python was used toward the calculation of the FFT coefficients for all the gestures. Raw and FFT IMU data are then stacked to form 3D matrices, both for the training and testing data sets. Both sets are also standardized, with the testing data set standardized in reference to the training data set statistics. In other words, the data are transformed to have a mean of zero and a standard deviation of one across each feature. This is done in response to differing scales between the components of the IMU, particularly between the accelerometer, gyroscope, and pitch angle. The dimensions of the training and testing matrices are thus 41808×W×14, and 844×W×14.

One dimensional output matrices are constructed to provide the desired output of the model, aligned with each hand movement in the testing and training matrices. The output of the prediction model is set to binary, designating a face touch to (1), or not a face touch to (0).

### 4.3 CNN-Based Prediction Model Architecture

**FIGURE 4:**
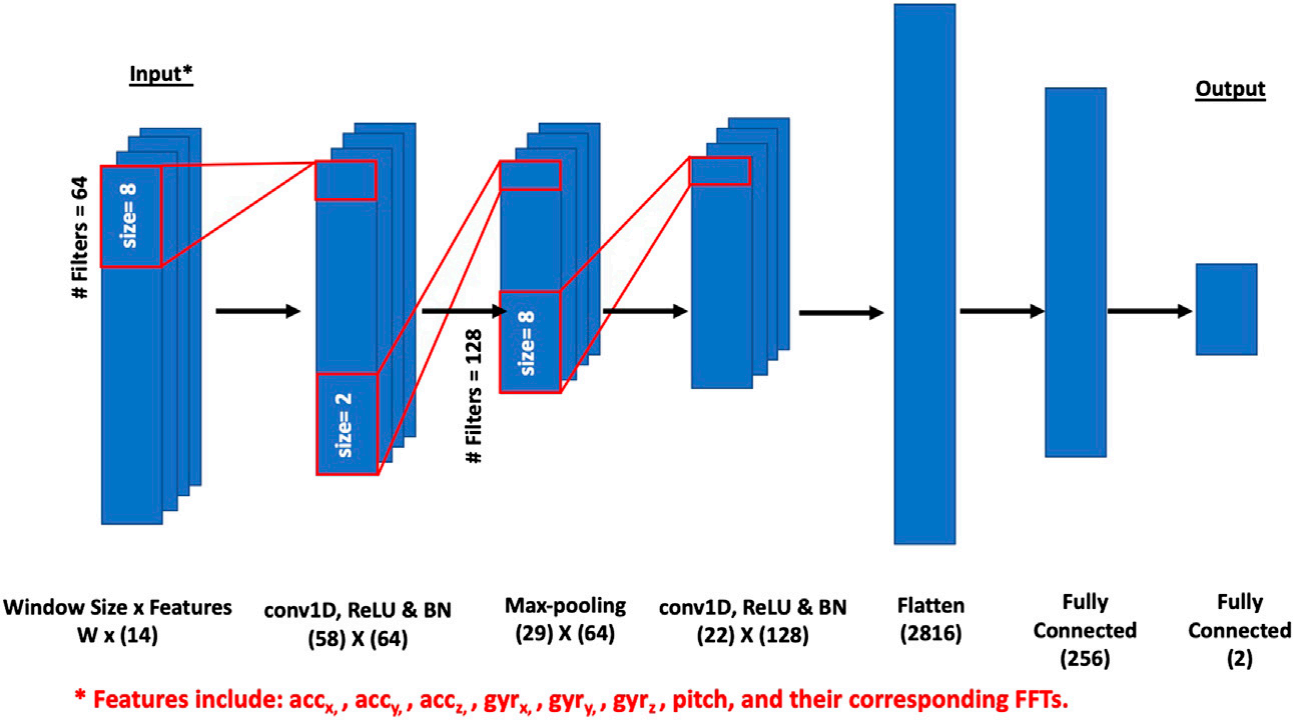
The architecture of the CNN-based prediction model. Note that W represents window size for the input matrix.

The input data used to train the model is arranged into a three-dimensional matrix: the first dimension represents the number of trials in the dataset, the second dimension is the time length of the gesture (each index represents a time step of 11 ms, in accordance to the 90.9 Hz IMU sampling rate), and the third dimension is the number of features. The number of features is defined by 6 degrees of freedom from the IMU (acceleration and gyroscope data), as well as the pitch angle value, and corresponding FFT coefficients to form a total depth of 14 features. These data are used to train and test the model, where first a convolution layer (conv1D) is applied, comprising 64 filters of kernel size 8. This is followed by a rectified linear unit (ReLu) activation function applied to the previous output, a batch normalization layer (BN), and a max-pooling layer with a pool size of 2. A dropout layer of value 0.8 is then applied. A second convolution layer is used, consisting of 128 filters also of kernel size 8, followed by another ReLu activation function. Batch normalization is utilized once more, along with a dropout layer of value 0.9, after which the input at its current state is passed through a flatten layer. Finally, two fully connected layers separated by a third dropout layer of value 0.8 are applied. The first fully connected layer has a dimensional unit of 256, with a softmax activation function, and the second has a dimensional unit of 2, with a ReLu activation function. The last fully-connected layer outputs two probabilities, one for each class (Not a face touch, face touch). The architecture for the CNN-based prediction model is shown in Figure 4.

### 4.4 Training and Performance Measures

The model shown in Figure 4 was trained using a categorical cross-entropy cost function with a default learning rate of 0.001, batch size of 512, and 300 epochs. The model was optimized (weights adjustment) using Adam optimizer Kingma and Ba (2014) during the training process. Batch normalization layers (BN) were used after each of the convolutional layers which basically re-centers and re-scales the input data leading to a faster and more stable training process. To avoid overfitting and prevent co-adaptation of the network weights, a dropout ratio (0.8–0.9) was used in the model. This high dropout ratio proved to work well with our study due to the relatively limited dataset which makes the model more prone to overfitting. The training accuracy reached 96.2% with a loss of 0.1. Table 1 shows the normalized confusion matrix of the results. The trained model has a sensitivity of 0.929 and a specificity of 0.935. This 1D-CNN model was finalized after many optimization rounds for the different hyper-parameters including the number of layers, filters and dropout ratios. An accuracy of 87.89, 89.7, 87.31% was obtained for a model with 3, 4, and 5 1D-convolutional layers respectively and thus, a model with 2 layers proved to be more efficient. Reducing the dropout ration to 0.5 reduces the classification accuracy to 90%. Thus, an optimized ratio of 0.8 or 0.9 was used.

**TABLE 1:** Normalized confusion matrix of the face touching/not face touching classification.

True label	Predicted label	
	Not face touching	Face touching
Not face touching	0.97	0.03
Face touching	0.11	0.89

### 4.5 Results

**FIGURE 5:**
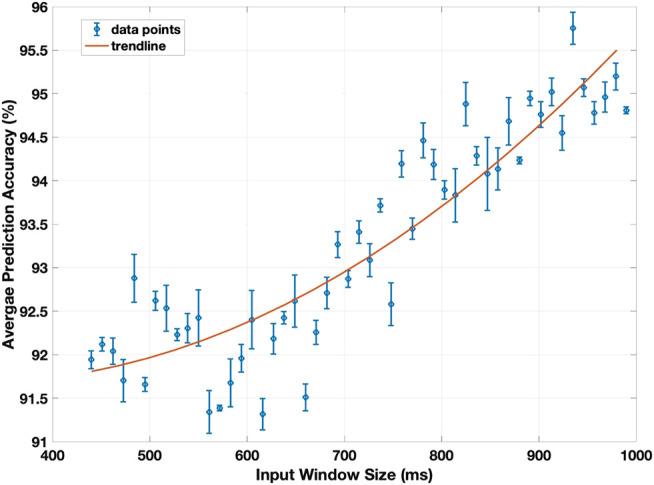
The prediction accuracy of the model against the input window size, averaged for the three conditions (sitting, standing, and walking). The input window size range is 440–990 ms, in increments of 11 ms, out of the 1,200 ms average time needed to touch the face.

With a focus on prediction rather than classification, the period for data collection in real time becomes a significant parameter to select. This window size limits the collection of data from the IMU during a hand movement. Figure 5 displays the resulting prediction accuracy as this window size is varied.

A shown in Figure 5, the prediction accuracy increases as the window size increases, with 95.7% test accuracy reported at around 935 ms. As expected, increasing the window size provides the CNN-based model with further information about the hand movement and thus improves the prediction accuracy. However, increasing the window sacrifices how fast a sensory feedback is presented to the user. When fully implemented, this prediction delay will also be extended by the inference time of the model. At a window size of 700 ms, the average inference time, in which the trained model classifies a single gesture, is 0.313 ms, and at a window size of 990 ms, is 0.446 ms. These values are small enough that they become negligible to the total time delay, effectively reducing time delay before prediction to depend only on window size.

The significance of this delay depends on the application of this device. In a case where it is crucial to keep the user from touching their face, a smaller window size will reduce the time delay before a prediction is made (and before the user can be warned sooner), thereby maximizing time for reaction. This increases the probability that the user will indeed be able to stop their hand movement and avoid touching their face. As is shown in Figure 5, the consequence of this is a reduced prediction accuracy, as reducing the size of the time window reduces the amount of information about the hand movement. With urgency being prioritized, however, false-positives along with ample time to react is still more favorable. In a case where the device is meant to act as a reminder and perhaps a non-essential deterrent, such as may be the case during the COVID-19 pandemic, a larger window size may be excused to achieve higher accuracy. Therefore, finding an optimum trade-off between response time and prediction accuracy through the window size depends largely on the application.

## 5 Sensory Feedback for Motor Control

A psychophysical experiment is presented to compare the effectiveness of three different sensory modalities, visual, auditory, and vibrotactile, as sources of feedback to stop the hand movement. The ability of a subject to stop their hand movement when confronted with sensory information is quantified by comparing the response time and success rate (percentage of times the user succeeds in avoiding face touching) for the three sensory modalities (p<0.05). Finally, a questionnaire was introduced to the participants at the end of the experiment to subjectively evaluate their quality of experience.

### 5.1 Participant

Thirty participants (15 female, 15 male, ages 25–50 years) are recruited for the experiment. None of the participants have any known sensorimotor, developmental or cognitive disorders at the time of testing. Written informed consent is obtained from all participants. The study is approved by the Institutional Review Board for Protection of Human Subjects at New York University Abu Dhabi (Project # HRPP-2020-108).

### 5.2 Experimental Setup

**FIGURE 6:**
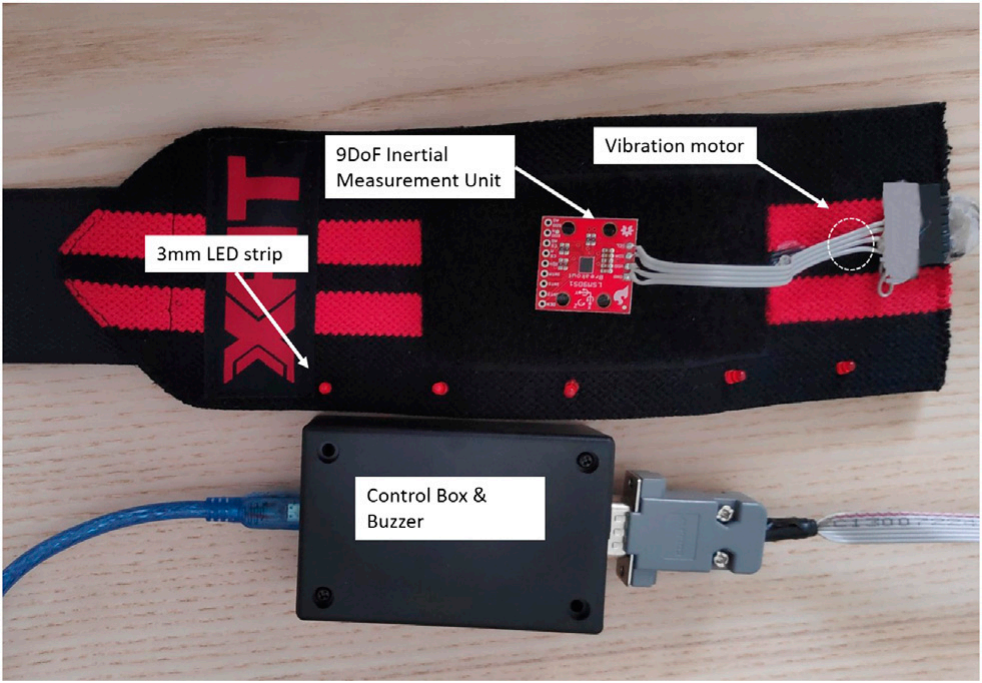
The wristband used in this study with all its components labeled.

A custom wristband is developed to provide the three sensory modalities, shown in Figure 6. A strip of five 3 mm LED’s is attached along the top face of the wristband to provide blinking visual feedback. On the bottom face of the wristband a coin type vibration motor is attached to provide vibrotactile feedback (Pico Vibe 310-177, Precision Microdrives vibration motor). At the middle of the top face, a 9 Degrees of freedom (DoF) IMU is placed in order to sense any movements (displacements and rotations) when the wristband is strapped on a hand. The wristband is connected to a control box which hosts the driving circuit of the vibration motor. A 1 kHz piezoelectric buzzer is utilized to provide auditory feedback. An ATMEGA328 microcontroller unit to control and acquire data from all of the aforementioned hardware components is used. The experimental setup is connected to a laptop through a serial connection over a USB cable.

Participants sit around 2 m in front of the experimenter where they could make unrestricted arm movements. Participants are asked to wear the wristband at their dominant hand and keep their hand in a resting position (on the table). The experimenter instructs the participants through the experiment verbally.

### 5.3 Experimental Task and Protocol

In this experiment, participants complete a face touching task. Participants are instructed to move their dominant hand to touch their face, during which the hand movement is occasionally interrupted by a stimulus cue that informs the subject to stop the movement in order to avoid touching the face. Each participant completes a total of 100 trials, with 30% of these trials provide sensory feedback while the other 70% of the trials have no sensory feedback and thus result in touching the face. Among the 30% with sensory feedback, 10% are visual, 10% are auditory, and 10% are vibrotactile. To minimize the learning effects that influence the response time, the trials are presented in a counterbalanced fashion.

A trial starts with the experimenter asking the participant to rest their dominant hand on the table with tactile sensing capability to detect the start of the hand movement. The experimenter instructs the participant to move their dominant hand and touch their face. During the hand movement, the sensory cue is applied at the wristband. The hand movement is analyzed based on the recorded IMU data. At the end of the trial, the experimenter prompts the participant to confirm whether they touched their face or not. The sensory stimulus is given at a random time during the movement. The visual stimulus is a blinking red light that shines around the wristband to make it clearly visible, and lasts for 500 ms. The auditory stimulus is a beeping sound at 1,000 Hz for 500 ms. The vibrotactile stimulus has a vibration frequency of 200 Hz and lasts for 500 ms. The intensity of vibration is set to be readily detected (defined as > 95% correct in stimulus detection). After completing the experiment, participants fill a questionnaire in order to evaluate their subjective experience.

The main quantification is the response time, which indicates how rapidly a subject can respond to a stimulus as a source of feedback and stop the ongoing hand movement. The response time is measured as the time between the onset of the sensory feedback stimulus and the time when the hand reaches a complete stop or reverses the direction of motion. The success rate—the percentage of times the participants succeeds to respond timely to the sensory feedback stimulus and avoid touching their face—is also recorded. The data are analyzed using repeated measures ANOVA (Analysis Of Variance) after confirming normal distribution (D’Agostino-Pearson normality test).

It is also worth noting that the experimental protocol followed COVID-19 preventive measures in terms of social distancing, symptom check for all participants, disinfection of study visit area before, and wearing personal protective equipment (surgical mask and gloves).

### 5.4 Results

**FIGURE 7:**
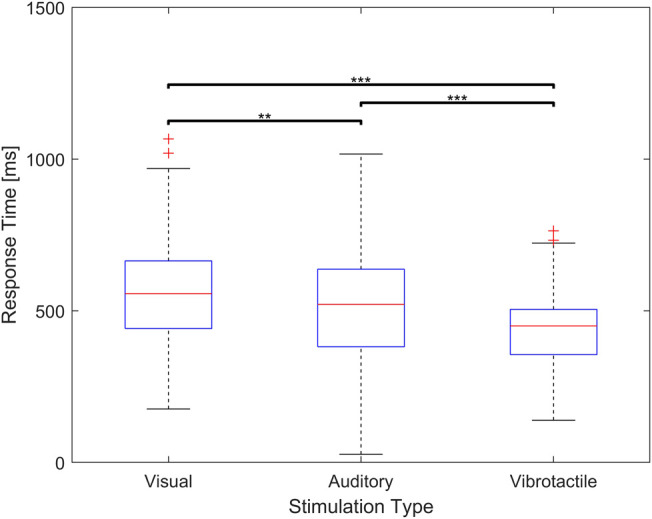
Response time for visual, auditory, and vibrotactile feedback. The middle red line of the blue box indicate a median value and the bottom and top edges indicate the 25th and 75th percentiles respectively (** means *p* < 0.05, *** means *p* < 0.01).

The average response time for vibrotactile stimulus is 427.3 ms with standard deviation of 110.88 ms. The average response time for visual stimuli is 561.70 ms with standard deviation of 173.15 ms. With regards to auditory stimulus, the average response time is 520.97 ms with standard deviation of 182.67 m. Response time to vibrotactile stimulus is found to be significantly shorter than that to auditory stimulus (*p* < 0.01) and visual stimulus (*p* < 0.01). Furthermore, the response time to auditory stimulus is found to be significantly shorter than that to visual stimulus (*p* < 0.05). A summary of these findings is shown in Figure 7.

**FIGURE 8:**
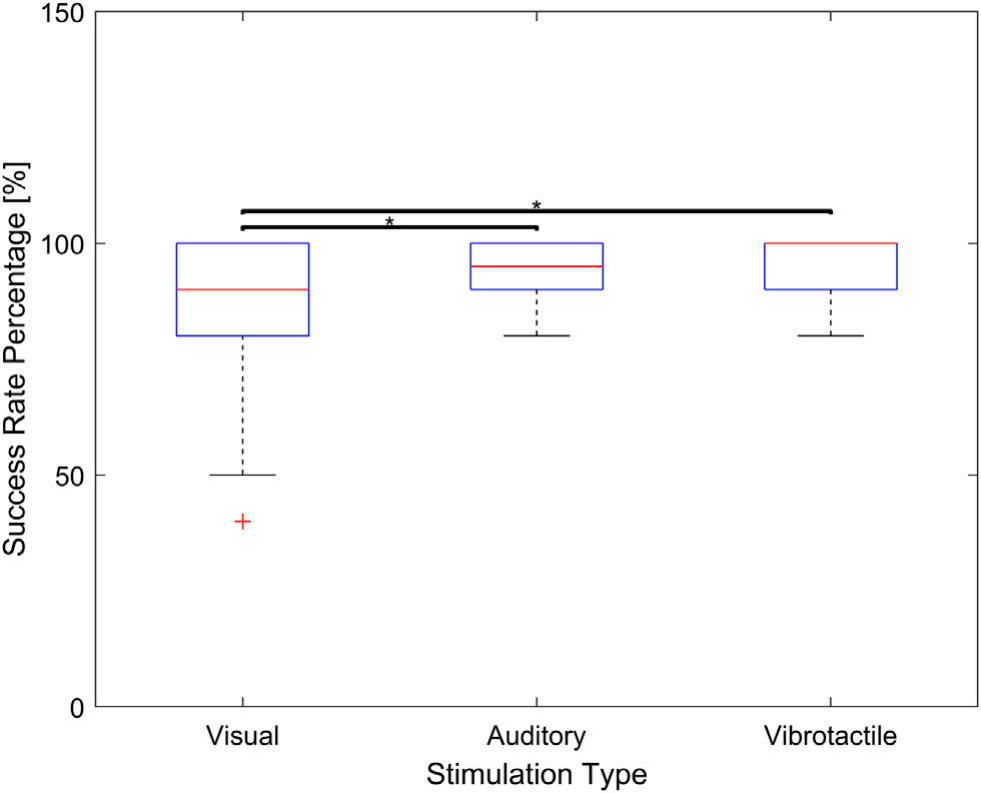
Success rate associated with visual, auditory, and vibrotactile feedback. The middle red line of the blue box indicate a median value and the bottom and top edges indicate the 25th and 75th percentiles respectively (* means *p* < 0.05).

Another important performance parameter to compare is the success rate. The average success rate for vibrotactile stimulus is found to be statistically larger than that of visual stimulus (*p* < 0.05). Furthermore, the average success rate for auditory stimulus is found to be statistically larger than that of visual stimulus (*p* < 0.05). However, there is no significant differences between vibrotactile stimulus and auditory stimulus (*p* = 0.07). Figure 8 shows the differences in success rate among the three groups.

The questionnaire is designed to capture the participant’s quality of experience. Participants are asked about their favorite modality for feedback, which modality provides the most pleasant experience, whether vibrotactile feedback creates any fatigue or discomfort, and the chance to provide any further feedback. As for preference, 25 participants (83.34%) selected vibrotactile as their favorite modality for feedback, 3 (10%) selected auditory feedback, and 2 (6.67%) preferred visual. 29 participants (96.67%) reported that they clearly perceived the vibrotactile stimulation. 28 participants (93.34%) selected vibrotactile feedback as the most pleasant among the three modalities. Finally, none of the participants reported significant fatigue or discomfort during the experiment.

## 6 Discussion

The CNN-based prediction model requires less than 550 ms of IMU data to predict face touching events with an accuracy greater than 92%. Furthermore, the sensory feedback experiment showed that around 427 ms is needed to stop the hand movement using vibrotactile feedback. Therefore, it will take less than a second from the start of the hand movement until complete stop. Meanwhile, our study suggests that the average time for a hand to reach and touch the face is 1,200 m. Therefore, the proposed system is capable of providing timely response to avoid face touching within less than 1 s. It is worth noting that there is a tradeoff between the prediction accuracy and the response time. In order to improve the prediction accuracy, the input window size must increase, which implies that it will take more time to stop the hand movement, which causes a decrease in chances to avoid face touching.

Another important factor is the relationship between prediction accuracy and practical usefulness of the system: an increase in the number of false positives would create unnecessary buzzing which may distract/annoy the user while an increase in false negatives would not prevent face touching entirely. Therefore, while the current prediction system is based solely on the IMU data, fusing other sensory modalities into the CNN-based model that are relevant to face touching and hand movement would significantly improve the prediction accuracy. For instance, gender, arm length, hand size, and age group may provide complementary information to improving the prediction accuracy. This involves recruiting a significantly larger number of participants to generate enough data points to train the model. In situations where camera data are available, such as when the user is sitting in front of a PC, computer vision approaches can be applied in order to fine-tune the model for improved performance.

A major source for false positives stems from the lack of information about the head posture in reference to the hand movement. Therefore, it would be interesting to augment the current CNN-based prediction model with the head position and/or orientation. With appropriate sensors or camera systems, the head posture can be continuously monitored and used as an auxiliary input to the prediction model to further improve the prediction accuracy. This is an interesting direction for future work. Furthermore, collecting hand movements that are likely to cause false positives (such as eating where the hand movement is very similar to that of face touching) and training the model with such data would significantly reduce the false positives.

Although the findings of the present study demonstrate the feasibility of developing a system to avoid face touching, a few limitations should be mentioned. First, the dataset utilized to train the CNN-based prediction model is rather limited. A larger dataset improves the prediction accuracy, including false positives and negatives, which allows for a reduced window size and improved system response. Furthermore, running the CNN model is computationally expensive. Therefore, the inference about the prediction of face touching may have to be performed on a computationally powerful machine such as a smart phone or even the cloud. This adds further delays to the overall system response. Additionally, the current study focused on preventing face touching through the dominant hand. It might be desirable for several applications to avoid touching the face with both hands, and thus evaluating the performance of the system while tracking both hands is necessary for such applications.

Another very important limitation is that the collected IMU data were pre-segmented such that each trial is known to have a single hand gesture. As the IMU signals are continuous streaming data, a sliding window must be used to segment the raw data to individual pieces in real time, each of which is the input of the CNN model. The length and moving step of the sliding window are hyper-parameters that need to be carefully tuned to achieve satisfactory performance. This problem is present not only in tasks that require constant gesture recognition, but also in other fields such as continuous speech recognition. Finally, the participants’ behavior or activities could modulate the hand movement and thus may impact the accuracy of the prediction model. More data must be collected while participants are engaged in various activities/behavior in order to enhance the resilience of the classifications against users’ activities/behavior.

## 7 Conclusion

This paper presented a system that utilizes IMU data to predict hand movement that results in face touching and provide sensory feedback to stop the hand movement before touching the face. A 1D-CNN-based prediction model, capable of automatically extracting temporal features of the IMU data through 1D-CNN filters, was developed and trained with IMU data collected from 4,800 trials recorded from 40 participants. Results demonstrated a prediction accuracy of more than 92% with less than 550 ms of IMU time series data. Compared to visual and auditory modalities, it was found that vibrotactile feedback results in statistically faster response, better success rate, and improved quality of user experience.

As for future work, it is of an importance to evaluate the combined prediction/response system as a whole in a realistic experimental environment (while performing everyday life activities). Furthermore, the authors plan to develop a light-weight CNN-based prediction model that optimizes computational power in order to run the prediction model on a wearable device (with limited computational power). Improving the dataset by collecting more data can immensely improve the model training and performance. In particular, collecting data from tasks that exhibit similar hand movements to face touching but do not involve face touching (such as eating) will improve the system robustness, particularly against false positives.

## Data Availability

The dataset used to train the CNN-based prediction model can be found in the Applied Interactive Multimedia research lab directory using this link: https://drive.google.com/drive/folders/13ffsGBzL_6-5bmFgIJhdLiVkC3VWIs3A.

## References

[B1] Al-Shamayleh A. S. Ahmad R. Abushariah M. A. M. Alam K. A. Jomhari N. (2018). A systematic literature review on vision based gesture recognition techniques. Multimed. Tools Appl. 77, 28121–28184. 10.1007/s11042-018-5971-z

[B2] Arrabito G. R. Mondor T. A. Kent K. J. (2004). Judging the urgency of non-verbal auditory alarms: a case study. Ergonomics 47, 821–840. 10.1080/0014013042000193282 15204277

[B3] Barroso F. Freedman N. Grand S. (1980). Self-touching, performance, and attentional processes. Percept. Mot. Skills 50, 1083–1089. 10.2466/pms.1980.50.3c.1083

[B4] Bate K. S. Malouff J. M. Thorsteinsson E. T. Bhullar N. (2011). The efficacy of habit reversal therapy for tics, habit disorders, and stuttering: a meta-analytic review. Clin. Psychol. Rev. 31, 865–871. 10.1016/j.cpr.2011.03.013 21549664

[B5] Calhoun G. Draper M. Ruff H. Fontejon J. Guilfoos B. (2003). “Evaluation of tactile alerts for control station operation,”in Proceedings of the human factors and ergonomics society Annual Meeting, Denver, Colorado, October 13–17, 2003 (Los Angeles, CA: SAGE Publications Sage CA). 47, 2118–2122.

[B6] Chen Z. Huang W. Lv Z. (2017). Towards a face recognition method based on uncorrelated discriminant sparse preserving projection. Multimed. Tools Appl. 76, 17669–17683. 10.1007/s11042-015-2882-0

[B7] Christian M. Uyanik C. Erdemir E. Kaplanoglu E. Bhattacharya S. Bailey R. (2019). “Application of deep learning to imu sensor motion,”in 2019 SoutheastCon, Huntsville, AL, April 11–14, 2019 (New York, NY: IEEE). 1–6.

[B8] Christofferson K. Yang R. (2020). FaceSpace–apple watch application. Tech. rep., DEVOPS, FaceSpace Application. Available at: https://facespace.app/ (Accessed March 9, 2021)

[B9] Chu D. K. Akl E. A. Duda S. Solo K. Yaacoub S. Schünemann H. J. (2020). Physical distancing, face masks, and eye protection to prevent person-to-person transmission of sars-cov-2 and covid-19: a systematic review and meta-analysis. Lancet 395, 1973–1987. 10.1016/S0140-6736(20)31142-9 32497510 PMC7263814

[B10] Corrales J. A. Candelas F. Torres F. (2008). “Hybrid tracking of human operators using imu/uwb data fusion by a kalman filter,”in 2008 3rd ACM/IEEE International Conference on Human-Robot Interaction (HRI). Amsterdams, Netherland, March 12–15, 2008 (New York, NY: IEEE).

[B11] D’Aurizio N. Baldi T. L. Paolocci G. Prattichizzo D. (2020). Preventing undesired face-touches with wearable devices and haptic feedback. IEEE Access 8, 139033–139043. 10.1109/ACCESS.2020.3012309 PMC8545332 34812343

[B12] Fu A. Yu Y. (2017). Real-time gesture pattern classification with imu data. FaceSpace Application. Available at: https://facespace.app/ (Accessed March 9, 2021)

[B13] Godlove J. M. Whaite E. O. Batista A. P. (2014). Comparing temporal aspects of visual, tactile, and microstimulation feedback for motor control. J. Neural Eng. 11, 046025. 10.1088/1741-2560/11/4/046025 25028989 PMC4156317

[B14] Hakkert A. S. Gitelman V. Ben-Shabat E. (2002). An evaluation of crosswalk warning systems: effects on pedestrian and vehicle behaviour. Transp. Res. Part F: Traffic Psychol. Behav. 5, 275–292. 10.1016/s1369-8478(02)00033-5

[B15] Harrigan J. A. (1985). Self-touching as an indicator of underlying affect and language processes. Soc. Sci. Med. 20, 1161–1168. 10.1016/0277-9536(85)90193-5 4023753

[B16] Huang J. Huang Z. Chen K. (2017). “Combining low-cost inertial measurement unit (IMU) and deep learning algorithm for predicting vehicle attitude,” in 2017 IEEE Conference on Dependable and Secure Computing, Taipei, China, August 7–10, 2017 (New York, NY: IEEE), 237–239.

[B17] Immutouch (2020). Immutouch wearable device. Available at: https://immutouch.com/ (Accessed September 03, 2020).

[B18] Jiang S. Lv B. Guo W. Zhang C. Wang H. Sheng X. (2017). Feasibility of wrist-worn, real-time hand, and surface gesture recognition via semg and imu sensing. IEEE Trans. Ind. Inform. 14, 3376–3385. 10.1109/TII.2017.2779814

[B19] Jiang X. Merhi L.-K. Menon C. (2017). Force exertion affects grasp classification using force myography. IEEE Trans. Human-Machine Syst. 48, 219–226. 10.1109/THMS.2017.2693245

[B20] Karia R. M. Ghuntla T. P. Mehta H. B. Gokhale P. A. Shah C. J. (2012). Effect of gender difference on visual reaction time: a study on medical students of bhavnagar region. IOSR J. Pharm. 2, 452–454. 10.9790/3013-0230452454[

[B21] Kemp B. J. (1973). Reaction time of young and elderly subjects in relation to perceptual deprivation and signal-on versus signal-off conditions. Develop. Psychol. 8, 268. 10.1037/h0034147

[B22] Kingma D. P. Ba J. (2014). Adam: a method for stochastic optimization. Preprint: arXiv:1412.6980.

[B23] Kwok Y. L. A. Gralton J. McLaws M.-L. (2015). Face touching: a frequent habit that has implications for hand hygiene. Am. J. Infect. Control 43, 112–114. 10.1016/j.ajic.2014.10.015 25637115 PMC7115329

[B24] Liu Y. Li Z. Liu Z. Wu K. (2019). “Real-time arm skeleton tracking and gesture inference tolerant to missing wearable sensors,”in Proceedings of the 17th Annual International Conference on Mobile Systems, Applications and Service, Seoul, Republic of Korea, June, 2019, 287–299.

[B25] Lv Z. (2020). Robust3d: a robust 3d face reconstruction application. Neural Comput. Appl. 32, 8893–8900. 10.1007/s00521-019-04380-w

[B26] Macchini M. Havy T. Weber A. Schiano F. Floreano D. (2020). Hand-worn haptic interface for drone teleoperation. Preprint: arXiv:2004.07111.

[B27] Macias A. E. De la Torre A. Moreno-Espinosa S. Leal P. E. Bourlon M. T. Ruiz-Palacios G. M. (2009). Controlling the novel A (H1N1) influenza virus: don't touch your face!. J. Hosp. Infect. 73, 280–281. 10.1016/j.jhin.2009.06.017 19699011

[B28] Manghisi V. M. Fiorentino M. Boccaccio A. Gattullo M. Cascella G. L. Toschi N. (2020). A body tracking-based low-cost solution for monitoring workers’ hygiene best practices during pandemics. Sensors 20, 6149. 10.3390/s20216149 PMC7663493 33138092

[B29] McMonnies C. W. (2008). Management of chronic habits of abnormal eye rubbing. Cont. Lens Anterior Eye 31, 95–102. 10.1016/j.clae.2007.07.008 18356094

[B30] Mueller S. M. Martin S. Grunwald M. (2019). Self-touch: contact durations and point of touch of spontaneous facial self-touches differ depending on cognitive and emotional load. PLoS One 14, e0213677. 10.1371/journal.pone.0213677 30861049 PMC6413902

[B31] Mummadi C. K. Leo F. P. P. Verma K. D. Kasireddy S. Scholl P. M. Kempfle J. (2018). Real-time and embedded detection of hand gestures with an imu-based glove. Inform. 5, 28. 10.3390/informatics5020028

[B32] Ng A. W. Chan A. H. (2012). “Finger response times to visual, auditory and tactile modality stimuli,” in Proceedings of the international multiconference of engineers and computer scientists, Kowloon, Hongkong, March 14–16, 2012. 2, 1449–1454.

[B33] Olivares A. Olivares G. Mula F. Górriz J. M. Ramírez J. (2011). Wagyromag: wireless sensor network for monitoring and processing human body movement in healthcare applications. J. Syst. Archit. 57, 905–915. 10.1016/j.sysarc.2011.04.001

[B34] Pawar T. Chaudhuri S. Duttagupta S. P. (2007). Body movement activity recognition for ambulatory cardiac monitoring. IEEE Trans. Biomed. Eng. 54, 874–882. 10.1109/tbme.2006.889186 17518284

[B35] Pisharady P. K. Saerbeck M. (2015). Recent methods and databases in vision-based hand gesture recognition: a review. Comput. Vis. Image Underst. 141, 152–165. 10.1016/j.cviu.2015.08.004

[B36] Rivera P. Valarezo E. Valarezo E. Choi M.-T. Kim T.-S. (2017). Recognition of human hand activities based on a single wrist imu using recurrent neural networks. Int. J. Pharma Med. Biol. Sci. 6, 114–118. 10.18178/ijpmbs.6.4.114-118

[B37] Sanders A. F. (1975). The foreperiod effect revisited. Q. J. Exp. Psychol. 27, 591–598. 10.1080/14640747508400522

[B38] Scott J. J. Gray R. (2008). A comparison of tactile, visual, and auditory warnings for rear-end collision prevention in simulated driving. Hum. Factors 50, 264–275. 10.1518/001872008x250674 18516837

[B39] Siddiqui N. Chan R. H. (2020). Multimodal hand gesture recognition using single iMU and acoustic measurements at wrist. PLoS One 15, e0227039. 10.1371/journal.pone.0227039 31929544 PMC6957149

[B40] Silva do Monte Lima J. P. Uchiyama H. Taniguchi R.-i. (2019). End-to-end learning framework for imu-based 6-dof odometry. Sensors (Basel) 19, 3777. 10.3390/s19173777 PMC6749526 31480413

[B41] Solomon S. S. Hill P. F. (2002). Emergency vehicle accidents: Prevention, reconstruction, and survey of state law. Tucson, AZ: Lawyers & Judges Publishing.

[B42] Thompson P. D. Colebatch J. G. Brown P. Rothwell J. C. Day B. L. Obeso J. A. (1992). Voluntary stimulus-sensitive jerks and jumps mimicking myoclonus or pathological startle syndromes. Mov. Disord. 7, 257–262. 10.1002/mds.870070312 1620144

[B43] Ward J. A. Lukowicz P. Troster G. Starner T. E. (2006). Activity recognition of assembly tasks using body-worn microphones and accelerometers. IEEE Trans. Pattern Anal. Mach. Intell. 28, 1553–1567. 10.1109/tpami.2006.197 16986539

[B44] WHO (2020). World health organization. Avaliable at: https://covid19.who.int/ (Accessed June 09, 2020).

[B45] Yang C. Lv Z. (2020). Gender based face aging with cycle-consistent adversarial networks. Image Vis. Comput. 100, 103945. 10.1016/j.imavis.2020.103945

[B46] Zhao J.-L. Wu Z.-K. Pan Z.-K. Duan F.-Q. Li J.-H. Lv Z.-H. (2018). 3D face similarity measure by fréchet distances of geodesics. J. Comput. Sci. Technol. 33, 207–222. 10.1007/s11390-018-1814-7

